# Effectiveness and utilization of a cognitive screening program for primary geriatric care

**DOI:** 10.1186/s13195-024-01637-y

**Published:** 2025-01-17

**Authors:** David P. Salmon, Anna Malkina, Melanie L. Johnson, Christina Gigliotti, Emily A. Little, Douglas Galasko

**Affiliations:** 1 Department of Neurosciences, University of California, San Diego, La Jolla, CA 92093-0948 USA; 2 Shiley-Marcos Alzheimer’s Disease Research Center, University of California, San Diego, USA; 3 Department of Medicine, University of California, San Francisco, USA

**Keywords:** Cognition, Dementia, Memory, Screening, MCI, Primary Care, Cognitive Screening, Memory Screening

## Abstract

**Background:**

Effective detection of cognitive impairment in the primary care setting is limited by lack of time and specialized expertise to conduct detailed objective cognitive testing and few well-validated cognitive screening instruments that can be administered and evaluated quickly without expert supervision. We therefore developed a model cognitive screening program to provide relatively brief, objective assessment of a geriatric patient’s memory and other cognitive abilities in cases where the primary care physician suspects but is unsure of the presence of a deficit.

**Methods:**

Referred patients were tested during a 40-min session by a psychometrist or trained nurse in the clinic on a brief battery of neuropsychological tests that assessed multiple cognitive domains. Short questionnaires covering subjective cognitive complaints, symptoms of depression, and medical history were also administered. Results were conveyed to a dementia specialist who reviewed them and returned their judgement of the validity of the cognitive complaint to the primary care provider. Retrospective medical records review was carried out for a random (stratified) half of the sample to determine how screening results were utilized. Screening tests were repeated after two years in a subset of 69 patients.

**Results:**

The 638 patients screened (mean age = 75.9 years; mean education = 14.9 years; 58% women) were classified by screening as having normal cognition (*n* = 177), depression (with possible cognitive changes; *n* = 115), mild cognitive impairment (MCI; *n* = 107), or dementia (*n* = 239). Classification accuracy was shown by high agreement with the eventual clinical diagnosis in the medical record (69%; Cohen’s Kappa = .38; *p* < .001; 77% if MCI and dementia were collapsed; Cohen’s Kappa = .58; *p* < .001) and longitudinal decline in cognitive test scores only in those initially classified as having MCI or dementia. Medical records documented discussion of screening results with the patient in 69% of cases (80% if MCI or dementia was detected) and often referral to a specialist (62%), new brain imaging (54%), or change in medication (58%) when screening indicated potential cognitive impairment.

**Conclusion:**

The cognitive screening program was well accepted by primary care providers as an efficient and effective way to evaluate concerns about cognitive decline in older adults.

**Supplementary Information:**

The online version contains supplementary material available at 10.1186/s13195-024-01637-y.

## Introduction

Approximately 6.2 million Americans aged 65 and older are currently living with Alzheimer’s disease (AD) and this number may rise to nearly 14 million by 2060 [1]. The vast majority of these individuals will initially present with complaints of cognitive decline in primary care settings. It is essential that those providing primary geriatric care have a way to accurately and effectively detect cognitive impairment associated with AD, particularly now that there are new treatment options that can alter the course of the disease [2, 3]. Primary care providers must also be aware of cognitive impairment that can negatively impact medical care of geriatric patients by impeding doctor-patient communication, reducing a patient’s understanding of their medical condition, or interfering with treatment adherence [4].

The ability to effectively detect cognitive impairment in the primary care setting is limited by several factors. First, most physicians are not formally trained in cognitive assessment beyond the bedside mental status examination. While this level of assessment is sufficient for detecting frank cognitive impairment (e.g., dementia), most mental status examinations are not effective at detecting mild cognitive impairment (MCI) that may be an early manifestation of AD [5]. Second, there is a lack of time to conduct detailed objective cognitive testing in the busy clinic setting. The average patient visit with a primary care physician is less than 20 min and approximately six different health topics are covered per visit [6, 7], leaving little time to conduct even minimal formal cognitive testing. Finally, there are few well-validated cognitive screening instruments that can be administered and evaluated quickly without expert supervision in the primary care setting [8, 9]. These screening instruments are often not sensitive to MCI and may be influenced by confounding behavioral variables (e.g., depression, apathy) that complicate their interpretation.

Given these limitations, we developed a model cognitive screening program to provide relatively brief, objective assessment of a geriatric patient’s memory and other cognitive abilities in those cases where the primary care physician suspects but is unsure of the presence of a deficit. In this screening program, brief objective cognitive testing is performed by a trained psychometrist located in the geriatric clinic one day a week. Results are conveyed to an off-site neuropsychologist or behavioral neurologist with expertise in dementia and AD who reviews them and determines the presence or absence of cognitive impairment and possible contributory factors (e.g., possible depression). The determination is returned to the primary care physician within one to two weeks so they can use it within the context of all other medical information about the patient that is known to them. The screen does not provide a diagnosis but provides information about the validity and scope of cognitive impairment to help the primary care physician make a diagnosis or design further assessment (including possible specialty referral) and a plan of care. The cognitive screening procedure is completed in approximately 40 min and includes standardized objective tests of memory (immediate and delayed recall), visual attention, mental flexibility, visuospatial function, language, and orientation, as well as screening questions for symptoms of depression, subjective memory complaints, major medical (e.g., head trauma) or psychiatric (e.g., depression) history, and current medication use. Demographic information (e.g., age, gender, years of education) is collected to contextualize test interpretation.

The cognitive screening program was implemented for primary care physicians in the Family and Preventative Medicine Department and Geriatric Medicine Division of the UCSD Healthcare network. The following guidelines were established: (1) the target cohort was geriatric patients, aged 60 or over, who have a complaint about their memory or other aspect of cognition. The complaint may come from a family member or other knowledgeable informant, or cognitive impairment may be suspected by the primary care physician; and (2) the primary care physician (or other physician specialist) should be unsure that the complaint reflects a true, diagnosable cognitive deficit. Patients with frank, moderate-to-severe dementia should be readily detectable by the primary care physician [10] and are not appropriate for the cognitive screening program.

The present study examined the utilization of the screening program by primary care physicians and its impact on patient care. We determined whether or not the results and recommendations from screening were conveyed to the patient, and if they possibly contributed to decisions about the need for specialty referral or changes in treatment and care. We also examined longitudinal cognitive change over an average of two years in a subset of patients to evaluate the efficacy of the screening procedures for distinguishing cognitive impairment associated with a progressive disorder from normal age-related memory changes or depressed mood.

## Methods

### Participants

A total of 807 patients in the UCSD Healthcare network were referred by their primary care provider for brief, objective testing to screen for deficits in memory and other cognitive abilities. Referrals were generated from 187 different health care providers over a period of 5 years. Written informed consent to use their screening results in a research study on the efficacy and utilization of memory screening in the primary care setting was granted by 638 of those screened (79%), including 69 of 92 (77%) who had repeat screening an average of 23.6 months (s.d. = 12.0; range = 6–57 months) after initial screening. The final sample (*n* = 638) averaged 75.9 (s.d. = 9.2) years of age, 14.9 (s.d. = 3.2) years of education, and 58% were female (267 male/371 female). The final subsample with repeat screening (*n* = 69) averaged 75.2 years of age (s.d. = 7.9; range = 53–93 years), 15.3 years of education (2.9; range = 3–20 years) and 71% were female (20 male/49 female).

### Screening procedures, evaluation, and return of results

Participants were tested individually by a psychometrist or trained nurse in a quiet, well-lit room at one of two UCSD primary care clinics. A brief questionnaire was administered first that asked about demographic information (age, education, gender), history of head injury (yes/no), history of stroke (yes/no), current or history of depression diagnosis or treatment (yes/no), and current medications. Subjective cognitive decline (SCD) was then assessed with 5 yes/no questions about current presence of: 1) persistent memory difficulties, 2) difficulty finding words, 3) difficulty remembering people’s names, 4) misplacing belongings, and 5) difficulty completing complex tasks. A total SCD score (range 0–5) was created by assigning each positive response 1 point and summing across the five questions. Cognitive testing was then carried out using a brief battery of standardized neuropsychological tests that included the Mini-Mental State Exam (MMSE) [11], immediate and delayed recall conditions of the Wechsler Memory Scale-Revised Logical Memory Test (Story A only) [12], the Trail-Making Test (Parts A and B) [13], and all components of the 7-Minute Screen for dementia [14]: Benton Orientation Test, the 16-item Enhanced Cued Recall Test (Uncued and Total Recall), a Clock Drawing test, and a Category Fluency (“Animals”) test. Finally, the self-reported Beck Depression Inventory (BDI) [15] was completed. The entire screening session required approximately 40 min.

Each patient’s screening results were reviewed by a neuropsychologist and/or behavioral neurologist within 1 week of testing. Test scores were classified as normal or impaired compared to age and education appropriate normative data from the Mayo’s Older Americans Normative Studies (Logical Memory, Trail-Making Test Parts A and B) [16, 17] or the 7-Minute Screen validation study (Benton Orientation Test, Enhanced Cued Recall Test, Category Fluency Test, Clock Drawing Test) [14]. Age and education adjusted standard scores −1.5 or more below the normative mean for a given test were considered impaired. A cognitive domain (e.g., memory, language, executive function) was considered impaired if scores on one or more test measures within that domain were impaired. The overall 7-Minute Screen performance was further classified as indicative of “High,” “Moderate,” or “Low” probability of dementia according to a computerized scoring program that is based on the published algorithm [14] and provided with the test. BDI scores of 10 or higher (out of 63 possible points) were considered indicative of depressed mood [18]. After reviewing all data, expert consensus was reached between the neuropsychologist and behavioral neurologist to classify each participant as having normal cognition, depression without significant cognitive impairment, MCI, or dementia. MCI classification was assigned if cognitive impairment was judged to be primarily restricted to a single domain (evident on one or more score in that domain), and dementia classification was assigned if more than a single domain was involved.

When the review was complete, the MMSE score and one of the following statements was returned to the primary care physician to help them with diagnostic decision making and development of a plan of care based on their knowledge of the patient’s unique medical status:

1. *Mild memory impairment with good performance in other cognitive domains suggestive of Mild Cognitive Impairment (MCI) – a comprehensive assessment with further medical work-up and follow-up and/or referral to a specialist should be considered*. *MCI can be a prodromal stage of Alzheimer’s disease but also may result from conditions such as head trauma, stroke, alcohol, depression, or general medical illness. In people with low levels of education, the diagnosis of MCI requires caution.*
2. *No significant memory impairment, but mild impairment in other cognitive domains suggestive of Mild Cognitive Impairment (MCI) – a comprehensive assessment with further medical work-up and follow-up and/or referral to a specialist should be considered*. *MCI can be a prodromal stage of Alzheimer’s disease but also may result from conditions such as head trauma, stroke, alcohol, depression, or general medical illness. In people with low levels of education, the diagnosis of MCI requires caution*.
3. *Mild memory impairment with mild impairment in other cognitive domains. This may indicate early dementia*.* We recommend that the patient should undergo a comprehensive assessment to confirm these results and determine the likely underlying causes. Please note that patients with significant cognitive defects need to be reported to the Department of Motor Vehicles (DMV). Please review the Memory Screening results, and if they are consistent with a clinical impression of sufficient cognitive decline to affect driving, we recommend that you report the patient to the DMV.*
4. *Mild cognitive impairment and symptoms of depression on the Beck Depression Inventory – We suggest that you consider further work-up and treatment for depression and/or referral to a specialist. Vascular risk factors (e.g., hypertension, diabetes, increased cholesterol) or uncontrolled general medical problems should also be considered.*
5. *No evidence of significant impairment of memory or other areas of cognition, but symptoms of depression on the Beck Depression Inventory – We suggest that you consider further work-up and treatment for depression and/or referral to a specialist*.
6. *No evidence of significant impairment of memory or other areas of cognition – the patient’s symptoms most likely reflect brain changes accompanying normal aging*. *Reevaluation should occur in one year or sooner if cognitive symptoms worsen*.

### Retrospective medical records review

Retrospective medical records review was carried out an average of 21.5 months (s.d. = 4.8, range 3–24 months) after screening for 312 of the 638 participants (48.9%). The reviewed cohort was randomly selected with proportional sampling by date of screen and screening result (i.e., categorization of cognitive status). There was no significant difference between reviewed and non-reviewed subgroups in age (75.6 vs. 76.1 years [t(636) = −0.59; *p* = 0.55]), sex distribution (60.3% female vs. 56.1% female [χ^2^ = 1.11; *p* = 0.29]), MMSE scores (25.7 vs. 25.9 [t(635) = −0.46; *p* = 0.64]), BDI scores (10.5 vs. 9.9 [t(609) = −0.92; *p* = 0.36]), or SCD scores (3.4 vs. 3.3 [t(591) = −1.09; *p* = 0.28]) at time of screening. The reviewed subgroup had slightly more years of education than the non-reviewed group (14.6 vs. 15.2 years [t(635) = −2.68; *p* = 0.008]). Groups were similar in the distribution of patients categorized by memory screening as Normal Cognition (28.2% vs. 27.3%), Depression (14.1% vs. 21.8%), or MCI (14.7% vs. 18.7%), but there were more of those categorized as Dementia in the reviewed group (42.9% vs. 32.2%) [χ^2^ = 11.7; *p* = 0.009]. Medical records were reviewed in chronological order from the date of memory screening and covered up to 24 months. The following information was extracted: 1) was there a written note stating that the memory screening results had been discussed with the patient and/or the patient’s family (yes/no); 2) had the patient been referred to a specialist physician or specialty clinic (yes/no); 3) had there been a change in relevant medication (e.g., starting a dementia or depression pharmaceutical treatment, discontinuing a cognitively detrimental medication or drug) (yes/no and which medication); 4) had a new brain scan been obtained (yes/no and what type of scan); 5) had a dementia (e.g., MCI, Alzheimer’s disease; dementia) or depression diagnosis been made (yes/no and what diagnosis). Finally, any new psychiatric or neurological diagnosis was noted.

### Informed consent

The Cognitive Screening Program was carried out as a research project of the UCSD California Alzheimer’s Disease Center and the UCSD Shiley-Marcos Alzheimer’s Disease Research Center. Screening was free of charge to the patient. Informed consent to utilize the data for research purposes was sought prior to the screening assessment but the assessment was not contingent upon consenting to research. Data were included in the reported analyses only when written informed consent was obtained from the patient, or their caregiver, consistent with California State law. The research protocol was reviewed and approved by the Human Subjects Review Board at UCSD.

### Statistical analyses

Group comparisons of continuous demographic variables (e.g., age, education), BDI scores, SCD scores, and cognitive test scores were made with one-way Analysis of Variance (ANOVA) followed by post-hoc pair-wise group comparisons using Tukey’s Least Significant Difference (LSD) test (when the ANOVA result was significant). Group comparisons of categorical variables (e.g., sex, 7-Minute Screen classification) were made with χ^2^ tests. Concordance between diagnoses recorded in the medical record and cognitive screening classification or 7-Minutes Screen dementia probability classification were examined with Cohen’s Kappa statistic. Group (cognitive screening classification) X Time (evaluation 1 vs. evaluation 2) repeated measures ANOVA were used to examine differential change in cognitive test scores across classification groups. Concordance between cognitive screening classifications from evaluation 1 and evaluation 2 were examined with Cohen’s Kappa statistic. Statistical significance was set at *p* < 0.05 (two-tailed). Statistical analyses were conducted using SPSS version 29.0.2.0.

## Results

### Characteristics of patients referred for cognitive screening

The average MMSE score of the overall sample (*n* = 638) was 25.8 (s.d. = 4.0), average BDI score was 10.2 (s.d. = 8.0), and average SCD score was 3.3 (s.d. = 1.4). Almost all participants (94%) responded “yes” to at least one of the subjective cognitive decline questions, with “persistent memory difficulty” most common (81.2%), followed by “difficulty remembering names” (74.8%), “misplacing belongings” (64.2%), “word finding difficulty” (63.0%), and “difficulty with complex tasks” (49.8%). Most participants (86.8%) responded “yes” to more than one subjective cognitive decline question. A small minority of participants reported a prior head injury (16.6%) or history of a transient ischemic attack (TIA) or stroke (15.1%). More than 25% of participants reported current depression (26.3%) or a history of depression (22.4%), and 22.2% reported current use of antidepressant medication. Less than 3% of participants reported current use of a neuroleptic (2.4%), and 7.7% reported current use of an anticholinergic medication that could affect cognition. Only 4.8% of participants reported current use of a cholinesterase inhibitor to treat cognitive symptoms.

Participants were classified into four broad categories based on their screening results: Normal Cognition (*n* = 177; 27.7%), Depression (*n* = 115; 18.0%) without significant cognitive impairment, MCI (*n* = 107; 16.8%) with or without symptoms of depression, or Dementia with or without symptoms of depression (*n* = 239; 37.5%) (see Table 1). The classification groups differed in age [F(3, 634) = 34.9; *p* < 0.001] with the Dementia group older than all other groups, the MCI group older than the Depression and Normal Cognition groups, and the Normal Cognition group older than the Depression group (all p’s < 0.05). Education also differed [F(3, 633) = 10.3; *p* < 0.001] with the Dementia group less educated than the MCI group, and the Dementia and Depression groups less educated than the Normal Cognition group (all p’s < 0.05). There was a higher proportion of females in the Depression (67.0%) and Dementia (62.3%) groups than in the Normal Cognition (51.4%) and MCI (50.4%) groups [χ^2^ = 11.3; *p* < 0.01]. As expected, BDI scores differed [F(3, 610) = 63.2; *p* < 0.001] with higher scores for the Depression group than for the other three groups (all p’s < 0.05). SCD scores also differed [F(3, 592) = 7.4; *p* < 0.001] with higher scores for the Depression group than for the other three groups (all p’s < 0.05).

**Table 1:** Demographic, medical history, medication, and subjective cognitive decline (SCD) information by cognitive screening classification

	Normal Cognition	Depression	MCI	Dementia	
	(*n* = 177)	(*n *= 115)	(*n* = 107)	(*n* = 239)	*p*-value
Age	73.8 (8.4)	70.4 (10.0)	76.7 (8.2)	79.6 (8.1)	< .001
Education (years)	15.8 (3.0)	14.7 (3.2)	15.3 (2.9)	14.2 (3.3)	< .001
Sex (M/F)	86/91	38/77	53/54	90/149	< .01
Mini-Mental State Exam (MMSE)	28.4 (1.5)	28.1 (1.6)	27.1 (1.7)	22.3 (4.2)	< .001
Beck Depression Inventory (BDI)	8.2 (6.3)	18.3 (8.3)	7.5 (5.6)	8.8 (7.4)	< .001
Medical History (% yes)					
Head Injury	13.9	21.6	21.4	14.1	
Stroke or TIA	11.0	16.1	16.2	17.1	
Past Depression	20.7	41.6	16.8	17.0	
Current Depression	22.7	52.7	10.4	23.7	
SCD Total Score	3.3 (1.4)	3.9 (1.3)	3.0 (1.5)	3.2 (1.5)	< .001
SCD Items (% yes)					
Persistent Memory Difficulties	77.5	88.7	76.4	82.6	
Difficulty Finding Words	71.4	83.3	46.2	54.6	
Difficulty Remembering Names	76.6	85.2	72.1	69.5	
Misplaces Belongings	61.1	68.4	66.0	63.7	
Difficulty with Complex Tasks	45.3	59.5	36.5	54.6	
Current Medications (% yes)					
Cholinesterase Inhibitor	2.3	5.3	1.9	7.7	
Antidepressant	18.0	40.4	13.3	20.6	
Neuroleptic	1.7	3.5	1.9	2.6	
Anticholinergic	5.2	6.2	7.6	10.3	

Mean (s.d.) age, education, Mini-Mental State Exam score, Beck Depression Inventory score, and sex distribution are shown. The percentage of patients endorsing each medical history, medication, and subjective cognitive decline (SCD) question are shown

Classification groups differed significantly on all cognitive tests (by one-way ANOVA), as expected, given that test scores were used in determining group membership (see Table 2). Pair-wise group comparisons using Tukey’s Least Significant Difference (LSD) tests showed that the Dementia group scored worse than all other groups on all cognitive measures (all p’s < 0.05). The MCI group scored worse than both the Depression and Normal Cognition groups on the MMSE, Logical Memory Test, Enhanced Cued Recall Test, and Category Fluency Test (all p’s < 0.05). They also scored worse than the Normal Cognition group on Parts A and B of the Trail-Making Test (p’s < 0.05). The Depression group scored worse than the Normal Cognition group on the Immediate and Delayed Recall conditions of the Logical Memory Test (but not Percent Savings), Part B of the Trail-Making Test, and the Category Fluency Test (all p’s < 0.05). Classification groups differed in the proportion of individuals identified as having High, Medium or Low Probability of Dementia by the 7-Minute Screen [χ ^2^ = 342.1; *p* < 0.001]. Most in the Dementia group (76.5%) were identified as having High Probability of Dementia, whereas most in the MCI (84.0%), Depression (90.1%) and Normal Cognition (97.1%) groups were identified as having Low Probability of Dementia.

**Table 2:** Mean (s.d.) cognitive test scores and 7-minute screen dementia probability by cognitive screening classification

	Normal Cognition	Depression	MCI	Dementia	
	(*n* = 177)	(*n* = 115)	(*n* = 107)	(*n* = 239)	One-way ANOVA
Logical Memory Test					
Immediate	26.4 (6.5)	24.3 (7.9)	16.9 (6.7)	9.9 (6.0)	F(3, 633) = 244.4; *p* < .001
Delayed	21.5 (7.0)	19.4 (8.4)	8.6 (6.7)	3.3 (4.0)	F(3, 631) = 340.1; * p* < .001
Percent Savings (%)	80.0 (14.9)	77.1 (17.7)	46.6 (28.1)	27.1 (30.4)	F(3, 632) = 199.9; *p* < .001
Trail-Making Test A (sec.)	45.6 (20.2)	49.2 (21.1)	52.8 (17.9)	85.6 (37.3)	F(3, 621) = 88.4; *p* < .001
Trail-Making Test B (sec.)	106.4 (51.9)	139.8 (70.3)	145.9 (62.4)	251.3 (66.2)	F(3, 592) = 187.4; *p* < .001
7-Minute Screen Tests					
Benton Orientation	0.6 (3.2)	1.0 (2.2)	2.3 (8.7)	15.6 (25.8)	F(3, 636) = 38.9; *p* < .001
Enhanced Cued Recall	15.7 (0.7)	15.6 (0.8)	14.7 (1.9)	11.5 (4.0)	F(3, 625) = 115.7; *p* < .001
Clock Drawing Test	6.6 (0.7)	6.5 (0.9)	6.3 (1.0)	4.6 (2.0)	F(3, 628) = 96.0; *p* < .001
Category Fluency	19.4 (5.2)	17.5 (4.8)	15.8 (4.3)	10.2 (4.2)	F(3, 634) = 148.6; *p* < .001
7-Minute-Screen Classification					
“Low” Dementia Prob	97.10%	90.20%	84.00%	20.40%	
“Moderate” Dementia Prob	0.60%	3.60%	2.80%	3.40%	
“High” Dementia Prob	2.30%	6.30%	13.20%	76.50%	

### Utilization of cognitive screening results by primary care providers

Medical records review showed that cognitive screening results had been discussed by the primary care physician with the patient or the patient’s family for 69.2% of the overall sample (*n* = 312). This was especially true if screening had detected a potential problem (see Table 3). Results were discussed with more than 80% of patients classified as MCI or Dementia, and more than 60% of patients classified as Depression. A majority of those classified as MCI or Dementia had been subsequently referred to a specialist physician (e.g., neurologist) or clinic (62%), and/or for new brain imaging (54%). Approximately 50% of patients classified as Depression had been referred to a specialist physician or clinic. A change in medication that could affect cognition (e.g., Aricept prescribed, anticholinergic discontinued) was documented for 58.3% of patients classified as MCI or Dementia.

**Table 3:** Number (percentage) of patients with subsequent medical record documentation of various outcomes by screening classification

	Total	Normal Cognition	Depression	MCI	Dementia
Memory screening results were discussed with the patient or patient’s family	216 / 312 (69.2%)	40 / 88 (45.5%)	27 / 44 (61.4%)	42 / 46 (91.3%)	107 / 134 (79.9%)
Patient was referred to a specialist physician or clinic	159 / 312 (51.0%)	25 / 88 (28.4%)	22 / 44 (50.0%)	30 / 46 (65.2%)	82 / 134 (61.2%)
Relevant medication change (e.g., new medication, stop cognitively detrimental medication)	137 / 311 (44.1%)	17 / 84 (19.5%)	15 / 44 (34.1%)	28 / 46 (60.9%)	77 / 134 (57.5%)
New brain imaging was obtained	137 / 312 (43.9%)	24 / 88 (27.3%)	15 / 44 (34.1%)	19 / 46 (41.3%)	79 / 134 (59.0%)
New dementia-related diagnosis (e.g., Alzheimer’s Disease, MCI, dementia)	139 / 251^a^ (55.4%)	7 / 56 (12.5%)	4 / 37 (10.8%)	29 / 42 (21.4%)	99 / 116 (85.3%)
New depression diagnosis	63 / 251^a^ (25.1%)	22 / 56 (39.3%)	27 / 37 (73.0%)	7 / 42 (16.7%)	8 / 116 (6.9%)

^a^Only counted if any new diagnosis was documented in the chart

### Cognitive screening classification versus primary care physician’s clinical diagnosis

A clinical diagnostic impression was recorded for 251 of the 312 patients (80.4%) in the medical records review sample (see Supplementary Table 1, AdditionalFile1). The recorded clinical diagnosis was dementia for 115 patients (79 Alzheimer’s disease, 33 dementia, 3 dementia with depression), MCI for 24 patients (22 MCI, 2 MCI with depression), depression for 63 patients, and no cognitive impairment (i.e., normal) for 20 patients. Other diagnoses were noted for 29 patients (not included in subsequent analyses), including Parkinson’s disease (*n* = 7), ischemia or TIA (*n* = 7), brain tumor (*n* = 4), cerebrovascular accident (*n* = 3), alcoholism (*n* = 1), anxiety (*n* = 1), dyskinesia (*n* = 1), hallucinations (*n *= 1), hypothyroidism (*n* = 1), insomnia (*n* = 1), and seizure disorder (*n* = 1).

There was substantial concordance (69% agreement) between medical record diagnoses and cognitive screening classification as Normal Cognition, Depression, MCI, and Dementia (Cohen’s Kappa = 0.384; *p* < 0.001; see Table 4A). The overall agreement was even greater when MCI and Dementia categories were combined to reflect a general “cognitive impairment” category (overall agreement of 77%; Cohen’s Kappa = 0.577; *p* < 0.001). Those classified as Dementia by cognitive screening were usually clinically diagnosed with a dementia syndrome (86.1%), while those classified as MCI were usually diagnosed with either MCI (43.2%) or dementia (35.1%) (i.e., 78.3% agreement with a “cognitive impairment” diagnosis). The high percentage of those classified as MCI but subsequently diagnosed as dementia may reflect consideration of functional impairment by the primary care provider in the full diagnostic process. Those classified as Depression by cognitive screening were usually clinically diagnosed with depression, depressed mood, or anxiety (84.4%). However, a relatively high percentage of individuals classified as Cognitively Normal (i.e., not depressed) were also subsequently diagnosed with depression (47.8%). Overall, 27 out of 49 (55.1%) individuals who eventually received a clinical diagnosis of depression (without cognitive impairment) were detected during the cognitive screening process. Those classified as Normal Cognition by cognitive screening usually received a subsequent clinical diagnosis of normal cognition or depression with normal cognition (84.7%).

**Table 4:** Concordance between cognitive screening classification (panel A) or 7-minutes screen dementia probability classification (panel B) and clinical diagnosis reported in medical record of reviewed sample (*n* = 222). One individual with a reported clinical diagnosis of Depression and one with a reported clinical diagnosis of AD/Dementia did not complete all tests necessary to receive a 7-Minutes Screen dementia probability classification

Clinical Diagnosis in Medical Record	**Panel A**	Cognitive Screening Classification			
		Normal Cognition	Depression	MCI	Dementia
	Normal Cognition	17	1	1	1
	Depression	21	27	7	8
	MCI	1	1	16	6
	AD/Dementia	6	3	13	93
Clinical Diagnosis in Medical Record	**Panel B**	7-Minutes Screen Dementia Probability Classification			
		Low	Moderate	High	
	Normal Cognition	18	0	2	
	Depression	53	2	7	
	MCI	20	1	3	
	AD/Dementia	37	2	75	

There was also good overall concordance (69% agreement) between 7-Minute Screen dementia probability classification as “Low”, “Moderate” or “High” and medical record diagnoses (considering a clinical diagnosis of normal or depression to reflect “Low” dementia probability; Cohen’s Kappa = 0.342; *p* < 0.001; see Table 4B). However, the 7-Minute Screen classification was less accurate than full cognitive screening at identifying those with MCI, as only 4 of 24 (16.7%) who received a clinical MCI diagnosis were classified as “Moderate” or “High” likelihood of dementia by the 7-Minute Screen, whereas 22 of 24 (91.7%) who received a clinical MCI diagnosis were classified as MCI or Dementia by full cognitive screening. This was also true to a lesser degree for those who received a clinical diagnosis of dementia with only 77 of 114 (67.5%) classified as “Moderate” or “High” likelihood of dementia (one individual with a reported clinical diagnosis of AD/Dementia did not complete all tests necessary to receive a 7-Minute Screen dementia probability classification). In contrast, 93 of the 115 (81%) who received a clinical diagnosis of dementia were classified as Dementia by full cognitive screening.

### Longitudinal cognitive screening test performance

**Fig. 1:**
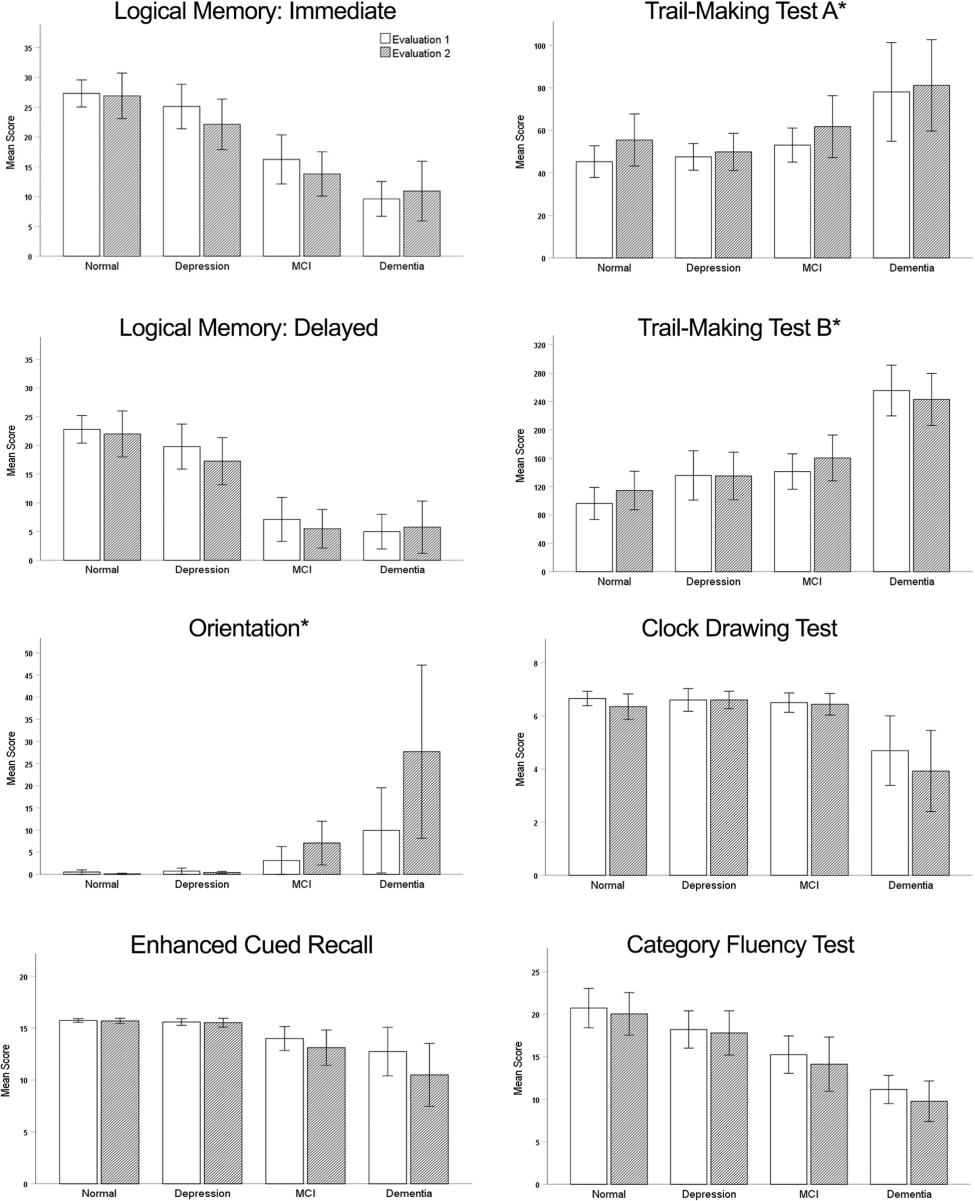
The mean scores achieved on each cognitive test at initial (evaluation 1) and follow-up (evaluation 2) screening evaluation by subgroups of patients who were classified as Normal Cognition, Depression, Mild Cognitive Impairment (MCI), or Dementia based on the initial cognitive screening evaluation. * Higher score = worse performance

We compared cognitive test scores achieved at the initial and two-year follow-up evaluations by a subgroup of patients (*n* = 69) who were initially classified as Normal Cognition (*n* = 25), Depression (*n* = 15), MCI (*n* = 16) or Dementia (*n* = 13), reasoning that if the initial classification was accurate there should be no change in test performance for those classified as Normal Cognition or Depression, and possible decline in test performance for those classified as MCI or Dementia. Classification groups did not differ significantly in length of inter-test interval, age, education, or sex distribution (all p’s > 0.05). A series of Group X Time (evaluation 1 vs. evaluation 2) repeated measures ANOVA for each cognitive test showed no significant main effect of Time or Group X Time interaction effect for immediate or delayed recall on the Logical Memory Test, Trail-Making Test Part A, Trail-Making Test Part B, Clock Drawing Test, or Category Fluency Test. There were, however, significant main effects of Time and Group X Time interaction effects for the Benton Orientation Test (Time: F(1,65) = 5.60; *p* = 0.021; Group X Time: F(3,65) = 3.38; *p* = 0.024) and the Enhanced Cued Recall Test (Time: F(1,63) = 11.47; *p* = 0.001; Group X Time: F(3,63) = 4.23; *p* = 0.009) due to decline over time in the Dementia and MCI subgroups but not in the Normal Cognition and Depression subgroups (see Fig. 1). As expected, there were significant effects of Group for all of the cognitive test measures (all p’s < 0.001). There was also a high degree of consistency in the cognitive screening classification received at evaluations 1 and 2 (see Supplementary Table 2, AdditonalFile1). Overall agreement after collapsing MCI and Dementia classifications was 75% (Cohen’s Kappa = 0.615; *p* < 0.001). Only 2 of 29 individuals classified as MCI or Dementia at evaluation 1 changed to Normal Cognition or Depression at evaluation 2 (i.e., a 7% “false positive” rate), while 7 of 40 individuals classified as Normal Cognition or Depression at evaluation 1 were classified as MCI or Dementia at evaluation 2 (i.e., 18% either “misclassification” or incident cognitive impairment).

It was noted in Fig. 1 that those initially classified as Dementia showed an unexpected improvement from evaluation 1 to evaluation 2, on average, on Logical Memory Test immediate and delayed recall and the Trail-Making Test Part B (although this improvement was not significant by paired t-tests; all *p* > 0.28). These apparent improvements were driven primarily by three participants who were classified as Dementia and had high BDI scores (all > 10) at evaluation 1. One of these participants was re-classified as MCI with depression at evaluation 2 (i.e., had received an initial “false positive” dementia classification at evaluation 1) and the other two remained classified as Dementia despite variability in test performance that may have been related to depression.

## Discussion

The cognitive screening program provided an efficient and effective way to objectively assess concerns of cognitive decline in geriatric patients in the primary care setting. When cognitive decline was suspected but uncertain at the initial medical evaluation, the primary care provider could simply direct the patient to cognitive screening and within days receive an expert opinion regarding the validity of the concern. This novel approach of treating cognitive screening as an outside laboratory test aligns with recommendations of the Interorganizational Summit on Population Health Solutions for Assessing Cognitive Impairment in Geriatric Patients [19] and provides an alternative to taking time to conduct screening during the busy clinic visit while allowing specialized expertise to be brought to bear [20]. Efficacy of the screening procedures for classifying patients as having normal cognition, possible depression, or cognitive impairment (i.e., MCI or dementia) was demonstrated by a high level of agreement with the comprehensive clinical diagnosis subsequently recorded in the medical record, and by longitudinal data that showed cognitive test scores declined after two years only in those initially classified as having MCI or dementia.

Cognitive screening results were highly utilized by primary care physicians. A review of patient medical records showed that cognitive screening results were discussed with the patient and documented in the medical chart of 69% of all those screened. When screening detected a potential cognitive problem, results were discussed with the patient and/or family member in a follow-up visit in approximately 78% of cases, and a new action (e.g., referral to a specialist, medication changes, neuroimaging) was taken in more than 79% of cases. These numbers are considerably higher than in previous studies which found that cognitive impairment was documented in the medical record for fewer than 25% of ambulatory adult general medical patients who actually had moderate to severe cognitive impairment [21], and only 54% of patients classified as having any degree of cognitive impairment in a large cohort study of women ≥ 75 years of age [20]. In the latter study, cognitive impairment was documented in only 23% of those with cognitive impairment without definite dementia. In contrast, impairment was documented in 91.3% of those classified as MCI in the present study. Lower levels of documentation in those classified as Normal Cognition or Depression in the present study may reflect failure to document when screening results simply reassured a patient of their normal aging or confirmed an existing depression diagnosis that had already been conveyed to the patient (psychiatric records are protected by privacy and were not available for our chart review).

The characteristics of patients referred by primary care physicians met the guidelines of the cognitive screening program. Of the 807 patients referred, 94.3% were age 60 or older, 94.3% had a cognitive complaint (i.e., scored ≥ 1 on the SCD scale), and 79.2% had an MMSE score ≥ 24 indicating questionable cognitive impairment. The prevalence of depression in those referred was high with 26.2% of participants reporting a current diagnosis of depression, 22.2% current use of antidepressant medication, and 22.4% a history of depression. More than 38% of the cohort scored above the established BDI cut-off for mild depression (i.e., ≥ 10 points) [18], although this score may be inflated by somatic symptoms in older patients. The prevalence of depression in our sample was higher than the 11.9% observed in a population-representative sample from the large Health and Retirement study (age ≥ 70) when symptoms of major or minor depression were combined with reported treatment for depression [22]. This may reflect a tendency for those with depression to have subjective cognitive decline [23, 24] which may lead to their over-representation for referral to cognitive screening. It should be noted, however, that subjective cognitive decline does not correlate with objective cognitive test performance in the current cohort [25].

The neuropsychological test battery used for cognitive screening was relatively brief, psychometrically robust, and covered a range of cognitive domains. The battery contained tests that effectively detected impairment and allowed patterns of deficits to be discerned that could support classification into our cognitive screening categories. While comparison of cognitive deficit profiles across our cognitive screening classification groups is somewhat circular given that the results were used in making the classifications, it is informative regarding which tests and deficit profiles carried the most weight in the decision process. Patients classified as Dementia scored worse than all other groups on all of the cognitive tests. Patients classified as MCI had a profile characterized by prominent deficits in all aspects of episodic memory (including rapid forgetting) with milder deficits in semantic memory and executive functions, and relatively preserved visuospatial abilities. This profile has been described in MCI with clinical, biomarker, or neuropathological verification of Alzheimer’s disease [26, 27]. Those classified as Depression had a profile of mild deficits that primarily affected executive functions such as set-shifting, psychomotor speed, and effortful retrieval, consistent with previous studies [28].

About 28% of participants were classified as cognitively normal by our cognitive screening procedures even though essentially all had some degree of SCD. Examination of Table 4A shows that, despite having SCD, 17 of 45 participants classified as Normal Cognition by screening were also classified as having normal cognition in the chart review diagnosis. These individuals may represent the “worried well” or may indicate that in some cases self-awareness of cognitive decline precedes a detectable decline in performance on standard cognitive tests used in our screening battery. There was insufficient longitudinal follow-up to determine if these cases eventually developed MCI or dementia. In addition, 21 of 45 participants classified as Normal Cognition by screening had a subsequent diagnosis of depression in the chart review (despite a normal BDI score at screening). This is consistent with previous findings that show SCD is often associated with depression [29]. Finally, 7 participants classified as Normal Cognition by cognitive screening later received a chart review diagnosis of MCI or dementia indicating they were “false negative” cases missed by screening.

The early detection of cognitive impairment in the primary care setting has gained importance since new disease modifying therapies for AD were approved for use in mildly symptomatic individuals with biomarker evidence of AD [30]. Primary care will play a critical role in determining when a patient with cognitive complaints should receive further evaluation and be referred for specialty care and potential treatment [31]. A survey of primary care physicians indicated that only 21% are highly confident they can correctly recognize neurocognitive disorder and only 20% have high confidence in their ability to interpret cognitive test results [32]. Primary care physicians have only moderate success in identifying cognitive impairment using only their clinical judgement [10, 33]. Widely-used brief standardized mental status examinations are inadequate as shown by more than 95% of those classified as MCI in the current study having MMSE scores in the normal range (i.e., ≥ 24). Thus, our cognitive screening program fills an important gap in care by providing accurate detection of mild cognitive decline that might trigger referral for specialty care and potential disease modifying treatment.

Several limitations of our study should be noted. First, the overwhelming majority of participants identified themselves as white and highly educated, reducing the generalizability of our results for a more diverse population. We will soon be able to address this important issue, however, as we have implemented a similar cognitive screening program with Spanish-language testing in primary care clinics that largely serve a socioeconomically disadvantaged Latino cohort near the U.S.-Mexico border. Second, screening was confined to patients with complaints of cognitive decline (i.e., symptomatic) or concerns expressed by others. Our results do not address the wider issue of general cognitive screening of all older individuals for early detection of unrecognized or unsuspected cognitive impairment [8]. A number of studies have described possible general screening methods [34–38], but whether or not to cognitively screen all individuals aged 65 or older as part of routine primary care remains controversial [39, 40] and is beyond the scope of our cognitive screening program. Finally, we need to determine how the cognitive screening process can assimilate rapidly emerging blood-based biomarkers of AD [41, 42]. When cognitive screening detects impairment, these minimally invasive biomarkers can help establish AD as the underlying cause, providing guidance on treatment with new disease modifying therapies [43]. Future research should address the most effective way of using these biomarkers for diagnosis and management of geriatric patients in the primary care setting.

## Supplementary Information

Additional file 1: Supplemental Table 1. Number of patients with each medical record clinical diagnosis by their cognitive screening classification. Supplemental Table 2. Concordance between cognitive screening classifications from the first (evaluation 1) and second (evaluation 1) evaluations in those with repeat screening after an average of approximately 2 years.

## Data Availability

The datasets used and/or analyzed during the current study are available from the corresponding author on reasonable request.

## Abbreviations

AD: Alzheimer’s disease
ANOVA: Analysis of Variance
BDI: Beck Depression Inventory
DMV: Department of Motor Vehicles
MCI: Mild cognitive impairment
MMSE: Mini-Mental State Examination
SCD: Subjective cognitive decline
TIA: Transient ischemic attack
