# Supplementary material for: Effectiveness and utilization of a cognitive screening program for primary geriatric care
Source: Alzheimers Res Ther. 2025 Jan 17;17:23. doi: 10.1186/s13195-024-01637-y (PMC11740611; doi:10.1186/s13195-024-01637-y)
Supplement: Supplementary file 1 — Additional file 1: Supplemental Table 1. Number of patients with each medical record clinical diagnosis by their cognitive screening classification. Supplemental Table 2. Concordance between cognitive screening classifications from the first (evaluation 1) and second (evaluation 1) evaluations in those with repeat screening after an average of approximately 2 years. [file 13195_2024_1637_MOESM1_ESM.docx]

Supplementary Table 1. Number of patients with each medical record clinical diagnosis (DX) by their cognitive screening classification.

Cognitive Screening Classification

Normal Cognition Depression MCI Dementia

(n=88) (n=44) (n=46) (n=134)

Medical Record DX

Normal Cognition 17 1 1 1

Alzheimer’s Disease 2 1 11 65

Dementia 4 1 2 26

Dementia (depress) 0 1 0 2

MCI 1 1 14 7

MCI (depression) 0 0 2 0

Depression 21 27 7 8

Anxiety 1 0 0 0

Insomnia 0 1 0 0

Hallucinations 0 0 1 0

Alcoholism 0 0 0 1

Cerebral Vascular Accident 2 0 0 1

Ischemia 2 1 1 2

Transient Ischemic Attack 1 0 0 0

Brain Tumor 1 0 2 1

Seizure Disorder 0 0 0 1

Parkinson’s Disease 2 3 1 1

Dyskinesia 1 0 0 0

Hypothyroidism 1 0 0 0

No Diagnosis Noted 32 7 4 18

MCI=Mild Cognitive Impairment.

Supplementary Table 2. Concordance between cognitive screening classifications from the first (evaluation 1) and second (evaluation 1) evaluations in those with repeat screening after an average of approximately 2 years (n=69).

| Cognitive Screening Classification: Evaluation 2 |  | Cognitive Screening Classification: Evaluation 1 | | |
| --- | --- | --- | --- | --- |
|  |  | Normal Cognition | Depression | MCI/Dementia |
|  | Normal Cognition | **15** | 4 | 1 |
|  | Depression | 4 | **10** | 1 |
|  | MCI/Dementia | 6 | 1 | **27** |
